# The role of basic health insurance on depression: an epidemiological cohort study of a randomized community sample in Northwest China

**DOI:** 10.1186/1471-244X-12-151

**Published:** 2012-09-20

**Authors:** Donghua Tian, Zhiyong Qu, Xiaohua Wang, Jing Guo, Fan Xu, Xiulan Zhang, Cecilia Lai-Wan Chan

**Affiliations:** 1 School of Social Development and Public Policy, Beijing Normal University, 19 Xinjiekouwai St, Beijing, China; 2 China Institute of Health, Beijing Normal University, 19 Xinjiekouwai St, Beijing, China; 3 Center on Behavioral Health, The University of Hong Kong, 5 Sassoon Road, Pokfulam, Hong Kong, China; 4 Department of Social Work and Social Administration, The University of Hong Kong, Pokfulam, Hong Kong, China

**Keywords:** Health insurance, Depression, Poverty

## Abstract

**Background:**

Little research has focused on the relationship between health insurance and mental health in the community. The objective of this study is to determine how the basic health insurance system influences depression in Northwest China.

**Methods:**

Participants were selected from 32 communities in two northwestern Chinese cities through a three-stage random sampling. Three waves of interviews were completed in April 2006, December 2006, and January 2008. The baseline survey was completed by 4,079 participants. Subsequently, 2,220 participants completed the first follow-up, and 1,888 completed the second follow-up. Depression symptoms were measured by the Center for Epidemiologic Studies Depression Scale (CES-D).

**Results:**

A total of 40.0% of participants had at least one form of health insurance. The percentages of participants with severe depressive symptoms in the three waves were 21.7%, 22.0%, and 17.6%. Depressive symptoms were found to be more severe among participants without health insurance in the follow-up surveys. After adjusting for confounders, participants without health insurance were found to experience a higher risk of developing severe depressive symptoms than participants with health insurance (7 months: OR, 1.40; 95% CI, 1.09-1.82; p = 0.01; 20 months: OR, 1.89; 95% CI, 1.37-2.61; p < 0.001).

**Conclusion:**

A lack of basic health insurance can dramatically increase the risk of depression based on northwestern Chinese community samples.

## Background

Major depressive disorder is a serious and recurrent disorder linked to diminished role functioning and quality of life, medical morbidity and mortality [1,2]. According to a recent study by the World Health Organization (WHO), the average lifetime and 12-month prevalence estimates of DSM-IV major depressive disorder were 14.6% and 5.5% in ten high-income countries and 11.1% and 5.9% in eight low- to middle-income countries [3]. China is in the period of "type-transforming", and people are facing various perplexities and problems. A large-scale mental health survey in 2009 revealed that the prevalence of mental disorders among the population aged 15 and older reached 17.5%, of which 6.7% had mood disorders, including depression [4]. The prevalence of depression among the senior population in rural areas of central China was 6% [5]. The rate was 24.8% (CES-D scores >16) among university students in Beijing, 8.9% of whom were severely depressed (CES-D scores >25) [6]. The high prevalence of mental disorders has become a serious public health problem and requires health policy support from the government.

Many studies have shown that access to health insurance improves physical health status. Examples include decreased death rates [7], shortened decision times required to receive emergency services [8], and increased utilization of medical services [9]. Studies have also shown that the lack of health insurance can worsen the health of breast cancer patients [10].

Few studies have explored the correlation between health insurance and mental health, and study conclusions are contradicting. Studies in the U.S. found that mandatory health insurance coverage of mental health services is not an effective means of improving mental health outcomes [11] or reducing the suicide rate, which is a primary indicator of mental health [12]. A contradictory result was found in a survey among 513 hospital inpatients in China, which revealed that those without health insurance had a higher risk of depression [13]. These differences may be due to the different cultures and health insurance systems.

In China, the universal and affordable health care system in urban cities is the Basic Health Insurance system, including the Urban Employee Basic Health Insurance Scheme (BHIS) and Urban Resident Basic Medical Insurance (URBMI). The BHIS was established in 1998 [14] and targets urban employees of the government and other public institutions, public and private enterprises and joint ventures with foreign companies [15]. The URBMI mainly covers those who are not officially employed, the elderly, students, and underage children. Since 2007, the URBMI has been operated under a pilot program in 79 cities [16]. In sum, the Basic Health Insurance system offers low-cost health insurance for urban residents. All the premiums are divided into two parts: social pooling and individual accounts. The enrolled employees and residents only need to contribute an affordable 2% of their monthly payroll to the pool fund to access basic health insurance, regardless of age, religion, sex and socioeconomic status [17]. Over 23.734 million people participated in the BHIS, and over 19.472 million people participated in URBMI by the end of 2010 [18].

Few longitudinal community sample studies have focused on the influence of health insurance on mental disorders, such as depression and few studies explore the relationship between BHIS and mental health in China. This study intends to fill this gap and provide information to identify the relationship between the risk of depression among the Chinese and their Basic Health Insurance coverage.

## Methods

### Study sites

This study is sponsored by the Project 985 fund of Beijing Normal University with approval from the Committee of Ethics of School of Social Development and Public Policy (SSDPP) at Beijing Normal University. In 2005, SSDPP launched a cohort study of a randomized community sample in Lanzhou City and Baiyin City in Gansu Province. The purpose of this project was to define the role of social assistance, including health insurance, medical services, and personal health as well as the interconnected relationships among them.

Gansu is a low-income region of western China. In recent years, the government established many preferential policies in the central and western regions to change the status quo. During the survey, the number of insured residents consistently increased. After the implementation of health insurance reform in the cities of Gansu, over 4.5 million people participated in the Basic Health Insurance system by the end of 2007 [19]. Lanzhou City, the capital of Gansu, is home to a population of 2,023,700, with a 2005 per capita disposable income of 8,529 yuan (US$1052.9) [20]. Baiyin City is 60 kilometers away from Lanzhou. By the end of 2005, its downtown population was 525,200, with a per capita disposable income of 7,928 yuan (US$978.8) [21].

### Participants

A three-stage cluster sampling process was used to select households to participate in the survey. During the first two stages, “probability proportional to size” sampling was used to select districts and communities based on their population size. During the third stage, 100 households were set to be equivalent in one sampling unit; the final number of households included in the sample was determined according to the number of sampling units of each community. Thus, using simple random sampling, the final sample includes a range of 100 to 300 households from each selected community. A total of 5,537 households from 32 communities throughout the two cities were eventually selected. One person who was 16 years of age or older in each household was asked to answer questions related to depression.

The baseline depression assessment was completed in April 2006. A total of 4,079 people (72.7% of the original selected sample) completed the depression assessment. The mean age of participants was 47.5 years (min = 16.0, max = 90.4; SD = 13.1). A total of 2,160 participants were female (53.0%), and 231 (5.7%) were ethnic minorities. When considering the educational status, 54.9% were junior high school graduates or lower, 33.9% were senior high school graduates, and 11.1% were college graduates or above.

The first follow-up survey was completed in December 2006, when 2,220 participants completed the depressive symptom assessment. This yielded a follow-up rate of 54.4%. Compared with those not interviewed in the first follow-up, the participants who completed the depression assessment had no significant differences in terms of city of residence, nationality, health insurance enrollment status, or CES-D scale scores. However, the participants did have several noticeable differences in characteristics. The participants who were not included in the first follow-up were younger (mean age of 45.4 years) and more likely to be female (54.7% vs. 51.5%). They had less than primary school education level (16.9% vs. 20.0%), were less likely to be Dibao families (families receiving the minimum living standard subsidy, 30.1% vs. 37.0%), and were less likely to be widowed or divorced (7.0% vs. 18.3%).

In January 2008, 1,888 participants completed the second follow-up depressive symptom assessment. The follow-up rate reached 46.3% of baseline, which included baseline participants who chose not to participate in the first follow-up but returned for the second follow-up. There were no significant differences in nationality, education status, and the baseline CES-D scale scores between the samples of participants who were followed up and those who had not. However, those participants who were not followed up were younger (mean age of 46.3 vs. 50.0 years), more likely to be female (59.3% vs. 45.6%), less likely to be receiving Dibao (31.4% vs. 36.6%), less likely to be widowed or divorced (7.4% vs. 19.8%), and less likely to have health insurance (37.7% vs. 42.7%). There are more sample details in Table 1.

**Table 1:** Baseline and two follow-up surveys sample size and participants’ characteristics

		**Baseline**	**7-month**			**20-month**		
			**interviewed**	**no**	**p**	**interviewed**	**no**	**p**
Sample		4079	2220	1859		1888	2191	
City, No. (%)	lanzhou	2961 (72.6)	1603 (72.2)	1358 (73.1)	.55	1572 (71.7)	1389 (73.6)	.19
	baiyin	1118 (27.4)	617 (27.8)	501 (26.9)		619 (28.3)	499 (26.4)	
sex, No. (%)	Male	1919 (47.0)	1076 (48.5)	843 (45.3)	.05	1027 (54.4)	892 (40.7)	<.001
	Female	2160 (53.0)	1144 (51.5)	1016 (54.7)		861 (45.6)	1299 (59.3)	
age, No. (%)	16-35	777 (19.0)	326 (14.7)	451 (24.3)	<.001	517 (23.6)	260 (13.8)	<.001
	36-45	1220 (29.9)	655 (29.5)	565 (30.4)		664 (30.3)	556 (29.4)	
	46-55	1048 (25.7)	617 (27.8)	431 (23.2)		525 (24.0)	523 (27.7)	
	56-65	674 (16.5)	388 (17.5)	286 (15.4)		324 (14.8)	350 (18.5)	
	> = 66	360 (8.8)	234 (10.5)	126 (6.8)		161 (7.3)	199 (10.5)	
Ethnicity, No. (%)	Han	3848 (94.3)	2107 (94.9)	1741 (93.7)	.08	1774 (94.0)	2074 (94.7)	0.34
	Minority	231 (5.7)	113 (5.1)	118 (6.3)		114 (6.0)	117 (5.3)	
education, No. (%)	primary or less	759 (18.6)	444 (20.0)	315 (16.9)	.001	362 (19.2)	397 (18.1)	0.25
	Junior high school	1482 (36.3)	825 (37.2)	657 (35.3)		705 (37.3)	777 (35.5)	
	High school	1384 (33.9)	735 (33.1)	649 (34.9)		625 (33.1)	759 (34.6)	
	College or above	454 (11.1)	216 (9.7)	238 (12.8)		196 (10.4)	258 (11.8)	
Dibao, No. (%)	yes	3283 (80.5)	821 (37.0)	559 (30.1)	<.001	691 (36.6)	689 (31.4)	<.001
	no	537 (13.2)	1399 (63.0)	1300 (69.9)		1197 (63.4)	1502 (68.6)	
marriage, No. (%)	Married	257 (6.3)	1741 (78.5)	1542 (83.0)		1460 (77.4)	1823 (83.2)	<.001
	Divorced	1286 (31.5)	407 (18.3)	130 (7.0)	.001	374 (19.8)	163 (7.4)	
	Never-married	1458 (35.7)	71 (3.2)	186 (10.0)		53 (2.8)	204 (9.3)	
employment, No. (%)	retired	1335 (32.7)	750 (33.8)	536 (28.8)	<.001	645 (29.4)	641 (34.0)	<0.01
	unemployed	1380 (33.8)	789 (35.5)	669 (36.0)		802 (36.6)	656 (34.7)	
	employed	2699 (66.2)	681 (30.7)	654 (35.2)		744 (34.0)	591 (31.3)	
health insurance, No. (%)	no	2445 (59.9)	1331 (60.0)	1114 (59.9)	.98	1081 (57.3)	1364 (62.3)	.001
	yes	1634 (40.1)	889 (40.0)	745 (40.1)		807 (42.7)	827 (37.7)	
CES-D Baseline, No. (%)	<16	1742 (42.7)	917 (41.3)	825 (44.4)	.08	974 (44.5)	768 (40.7)	.06
	16-20	766 (18.8)	416 (18.7)	350 (18.8)		405 (18.5)	361 (19.1)	
	21-25	684 (16.8)	373 (16.8)	311 (16.7)		364 (16.6)	320 (16.9)	
	≥26	887 (21.7)	514 (23.2)	373 (20.1)		448 (20.4)	439 (23.3)	

All surveying was conducted through face-to-face interviews. Prior to the interview, the study team delivered a letter to every selected family to inform them of the aim of the survey and their right to refuse to participate. Interviewers were required to show their documents and identification and introduce the interview aims prior to receiving permission to enter the participants’ houses.

### Measures

#### Main outcome measures

##### Depressive symptoms

The Center for Epidemiologic Studies Depression Scale (CES-D) Chinese edition [22] was used to assess depressive symptoms. This scale is the most widely used depression screening scale and is frequently applied to community-based studies. The Chinese version of the CES-D scale shows good reliability and validity across all ages in urban and rural population [23,24]. The Cronbach's Alpha reliability is 0.88 at baseline, 0.89 in the first follow-up, and 0.88 in the second follow-up.

Radloff has suggested that a score of 16 should be the cut-off point of the scale [25], while some studies have adopted 21 or 22 as the cut-off point [26]. Given the complexity of the cutoff point selection, this study applied the previous study methodology that defined four groups of depressive symptoms: (1) little or no symptoms of depression (CES-D Scale score <16), (2) mild depressive symptoms (16–20), (3) moderate depressive symptoms (21–25), and (4) severe depression (≥ 26) [27-29].

#### Exposurxe variables

##### Health insurance

The questionnaire asked participants to identify up to three types of health insurance in which they were enrolled. The available choices included all Chinese health insurance options. The results were recorded using a two-category variable: having no insurance or having at least one type of health insurance. To consider the change of insurance status throughout the three stages of the surveys, the variable was further subdivided into four categories: (1) having no health insurance in both the baseline and follow-up surveys, (2) having insurance at baseline but no insurance in the follow-up, (3) having no insurance at baseline but having insurance in the follow-up, (4) having insurance in both the baseline and follow-up surveys.

#### Confounders

##### Demographic and socioeconomic variables

A number of demographic and socioeconomic variables were included as potential confounders, including city, gender, age category (16–35, 36–45, 46–55, 56–65 and over 66), nationality (Han or Minority), educational status (primary school or below, junior high school, senior high school, college degree or above), employment (employed, retired, unemployed), and poverty, which is defined by Dibao (whether the family was receiving the minimum living standard subsidy).

##### Major life events

A number of major life events in the past year were included in the study: marriage, divorce, pregnancy, giving birth, being injured, death, being arrested, change in post and job, unemployment, significant income gain or loss, and change of place of residence. Each event was counted as one point, making 6 the highest score for major life events for all the participants. Finally, the score was grouped into three categories: 0, 1, and ≥ 2.

##### Health behaviors

Health behavior variables included smoking (more than one cigarette per day/never), drinking (more than once per week/never), and physical activities (frequent exercise: yes/no).

##### Body mass index (BMI)

Was calculated as weight in kilograms divided by height in square meters (kg/m^2^) and recorded as a three-category variable (< 19, 19–25, ≥ 25).

##### Any occurrence of disease in the previous 4 weeks was included as a categorical variable

0 = No disease in the previous 4 weeks, 1 = New acute disease in the most recent 4 weeks, 2 = Acute disease beginning earlier than, but continuing into, the most recent 4 weeks; and 3 = Chronic disease.

### Interviewers and training

The interviewers were all senior university students and first-year graduate students majoring in the social sciences. The interviewers spoke fluent Mandarin and mastered the northern accent, which made it easy for them to talk with the interviewees. All interviewers received a three-day training on survey principles, procedures, index, and definitions, and they also were required to interview each other to test their understanding of the interview. Each interviewer had to complete two pilot interviews in non-selected communities. Interviewer leaders were responsible for interview supervision of the interviews and regular questionnaire review. Upon identification of a problem, the interview leaders asked the interviewers for any corrections and explanations, as necessary.

### Statistical analyses

The SPSS statistical package (PASW 17.0; SPSS Inc., 2009-3-11) was used for data analysis. First, we employed a chi-squared test to analyze whether depression was significantly related to any of the variables reflecting demographics, family socioeconomic status, life events, health behaviors, and health status. We calculated the relationship between the above variables in the baseline interview and depression in all three surveys. Second, we used ordinal regression to examine the impact of health insurance enrollment status on depression in the 7-month and 20-month follow-up interviews. Third, we used the weighted data and ordinal regression to estimate depression prevalence. Because the sample included two family types (Dibao families and non-Dibao families), the weight also includes two types. One type was the weight of participants from Dibao families, which was the total population of Dibao families in the city divided by those included in the sample. Similarly, another weight was the total population of non-Dibao families in the city divided by those included in the sample. Finally, the list-wise approach was employed to deal with missing data in all statistical analyses.

## Results

### Descriptive analysis

The participant's health insurance status are presented in Table 2. In the baseline survey, 1,630 participants (40.0%) were enrolled in at least one health insurance or medical subsidy scheme, including 1,384 people with one insurance package, 234 with two, and 12 with three. Among the participants who had at least one type of health insurance, 1,547 (94.9%) were enrolled in the BHIS^a^. Given the high rate of this specific type of insurance, it was impossible to examine the influence of different insurance programs. Taking the sample weight into consideration, 48.7% (95% confidence interval [CI], 46.2-49.8) of the participants were insured.

**Table 2:** Descriptive characteristics of study population by health insurance status in two wave follow-up interviews

		**7 months later**				**20 months later**			
		**Non-insurance**	**Insurance to none**	**Non to insurance**	**Insurance**	**Non-insurance**	**Insurance to none**	**Non to Insurance**	**Insurance**
**Sample**		**1170 (52.7)**	**162 (7.3)**	**164 (7.4)**	**724 (32.6)**	**445 (23.6)**	**155 (8.2)**	**636 (33.7)**	**652 (34.5)**
city, No. (%)	Lanzhou	896 (55.9)	117 (7.3)	133 (8.3)	457 (28.5)	366 (26.3)	127 (9.1)	505 (36.4)	391 (28.1)
	Baiyin	274 (44.4)	45 (7.3)	31 (5.0)	267 (43.3)	79 (15.8)	28 (5.6)	131 (26.3)	261 (52.3)
sex, No. (%)	Male	467 (43.4)	83 (7.7)	79 (7.3)	447 (41.5)	205 (20.0)	90 (8.8)	289 (28.1)	443 (43.1)
	Female	703 (61.5)	79 (6.9)	85 (7.4)	277 (24.2)	240 (27.9)	65 (7.5)	347 (40.3)	209 (24.3)
age, No. (%)	16-35	219 (67.2)	18 (5.5)	20 (6.1)	69 (21.2)	71 (27.3)	16 (6.2)	109 (41.9)	64 (24.6)
	36-45	405 (61.8)	53 (8.1)	38 (5.8)	159 (24.3)	166 (29.9)	27 (4.9)	203 (36.5)	160 (28.8)
	46-55	306 (49.6)	38 (6.2)	55 (8.9)	218 (35.3)	111 (21.2)	49 (9.4)	172 (32.9)	191 (36.5)
	56-65	149 (38.4)	41 (10.6)	28 (7.2)	170 (43.8)	56 (16.0)	38 (10.9)	100 (28.6)	156 (44.6)
	≥66	91 (38.9)	12 (5.1)	23 (9.8)	108 (46.2)	41 (20.6)	25 (12.6)	52 (26.1)	81 (40.7)
nationality, No. (%)	Han	1096 (52.0)	157 (7.5)	156 (7.4)	698 (33.1)	409 (23.1)	145 (8.2)	597 (33.7)	623 (35.1)
	Minority	74 (65.5)	5 (4.4)	8 (7.1)	26 (23.0)	36 (31.6)	10 (8.8)	39 (34.2)	29 (25.4)
education, No. (%)	primary school or less	279 (62.8)	25 (5.6)	35 (7.9)	105 (23.6)	107 (29.6)	25 (6.9)	139 (38.4)	91 (25.1)
	Junior high school	470 (57.0)	56 (6.8)	62 (7.5)	237 (28.7)	171 (24.3)	48 (6.8)	256 (36.3)	230 (32.6)
	High school	358 (48.7)	58 (7.9)	52 (7.1)	267 (36.3)	145 (23.2)	55 (8.8)	201 (32.2)	224 (35.8)
	College or more	63 (29.2)	23 (10.6)	15 (6.9)	115 (53.2)	22 (11.2)	27 (13.8)	40 (20.4)	107 (54.6)
marriage, No. (%)	Married	851 (48.9)	132 (7.6)	125 (7.2)	633 (36.4)	306 (21.0)	127 (8.7)	453 (31.0)	574 (39.3)
	Divorced	264 (64.9)	23 (5.7)	34 (8.4)	86 (21.1)	115 (30.7)	26 (7.0)	162 (43.3)	71 (19.0)
	Never-married	54 (76.1)	7 (9.9)	5 (7.0)	5 (7.0)	24 (45.3)	2 (3.8)	20 (37.7)	7 (13.2)
employment, No. (%)	Retired	236 (31.5)	67 (8.9)	66 (8.8)	381 (50.8)	87 (13.6)	77 (12.0)	153 (23.9)	324 (50.5)
	Unemployed	655 (83.0)	38 (4.8)	41 (5.2)	55 (7.0)	246 (37.5)	28 (4.3)	332 (50.6)	50 (7.6)
	employed	279 (41.0)	57 (8.4)	57 (8.4)	288 (42.3)	112 (19.0)	50 (8.5)	151 (25.5)	278 (47.0)
Dibao, No. (%)	Yes	667 (81.2)	41 (5.0)	50 (6.1)	63 (7.7)	258 (37.3)	28 (4.1)	331 (47.9)	74 (10.7)
	No	503 (36.0)	121 (8.6)	114 (8.1)	661 (47.2)	187 (15.6)	127 (10.6)	305 (25.5)	578 (48.3)
Life events, No. (%)	No	906 (53.0)	123 (7.2)	123 (7.2)	556 (32.6)	334 (23.3)	121 (8.4)	488 (34.1)	489 (34.1)
	1	150 (50.8)	18 (6.1)	22 (7.5)	105 (35.6)	62 (23.7)	22 (8.4)	87 (33.2)	91 (34.7)
	≥2	114 (52.5)	21 (9.7)	19 (8.8)	63 (29.0)	49 (25.3)	12 (6.2)	61 (31.4)	72 (37.1)
smoke, No. (%)	No	857 (55.0)	117 (7.5)	118 (7.6)	466 (29.9)	309 (24.7)	108 (8.6)	450 (35.9)	386 (30.8)
	Yes	313 (47.3)	45 (6.8)	46 (6.9)	258 (39.0)	136 (21.4)	47 (7.4)	186 (29.3)	266 (41.9)
drink, No. (%)	No	1136 (54.0)	153 (7.3)	156 (7.4)	660 (31.4)	434 (24.4)	147 (8.3)	611 (34.3)	588 (33.0)
	Yes	33 (29.5)	9 (8.0)	7 (6.3)	63 (56.3)	11 (10.5)	8 (7.6)	24 (22.9)	62 (59.0)
Physical activity, No. (%)	yes	355 (45.8)	63 (8.1)	47 (6.1)	310 (40.0)	127 (19.5)	68 (10.5)	193 (29.7)	262 (40.3)
	No	815 (56.4)	99 (6.9)	117 (8.1)	414 (28.7)	318 (25.7)	87 (7.0)	443 (35.8)	390 (31.5)
BMI, No. (%)	<19	181 (66.8)	19 (7.0)	16 (5.9)	55 (20.3)	63 (27.3)	18 (7.8)	101 (43.7)	49 (21.2)
	19-24	801 (51.1)	113 (7.2)	117 (7.5)	538 (34.3)	296 (22.5)	109 (8.3)	435 (33.1)	476 (36.2)
	≥25	188 (49.5)	30 (7.9)	31 (8.2)	131 (34.5)	86 (25.2)	28 (8.2)	100 (29.3)	127 (37.2)
4-weeks disease, No. (%)	No	779 (50.6)	102 (6.6)	113 (7.3)	547 (35.5)	301 (22.9)	105 (8.0)	436 (33.2)	470 (35.8)
	acute disease in 4 weeks	67 (59.8)	10 (8.9)	10 (8.9)	25 (22.3)	29 (27.9)	5 (4.8)	32 (30.8)	38 (36.5)
	acute disease > 4 weeks	21 (63.6)	4 (12.1)	3 (9.1)	5 (15.2)	9 (31.0)	2 (6.9)	14 (48.3)	4 (13.8)
	Chronic	293 (56.6)	42 (8.1)	37 (7.1)	146 (28.2)	100 (23.5)	39 (9.2)	150 (35.2)	137 (32.2)

In the first follow-up interview, 1,894 (85.3%) had no change in their insurance enrollment status; 164 (7.4%) participants were newly enrolled in insurance; and 162 (7.3%) had lost their insurance. In the second follow-up, 1,097 participants (58.1%) had no change in their health insurance status; 636 participants (33.7%) were newly enrolled in insurance^b^; and 135 (8.2%) had lost their insurance.

The mean CES-D Scale depression scores for the study sample were 18.3 (SD = 9.3, range = 58.0) in the baseline survey, 17.7 (SD = 10.0, range = 60.0) in the first follow-up and 17.2 (SD = 9.2, range = 55.0) in the second follow-up. The correlation of the CES-D scale scores at baseline and in the first follow-up was 0.40 (p < 0.001), and the correlation between baseline and the second follow-up was 0.15 (p < 0.001).

Studies based in the Chinese cultural context suggest the original cutoff point of 16 has low positive predictive value [30] and may be too low for the Chinese [23]. Other studies indicate that a cutoff point of 21 has better positive predictive value for depression among the Chinese [31]. Studies in primary care settings of patients with higher levels of co-morbid medical illness have suggested that a cutoff of 21 has the best positive predictive value for major depression [32]. According to this standard, 33.1% (95% CI, 32.0-34.3) of the sample had depressive symptoms in the baseline survey. As used in previous studies, a cutoff point for severe depression of 26 results in 21.7% (95% CI, 20.5-23.0) of the sample with severe depressive symptoms in the baseline survey.

In the first follow-up, 31.2% (95% CI, 30.2-33.1) of the participants had depressive symptoms. The percentage of participants with severe depressive symptoms was 22.0% (95% CI, 20.3-23.8). In the second follow-up, 29.6% (95% CI, 27.6-31.7) of the participants had depressive symptoms. The percentage of participants with severe depressive symptoms was 17.6% (95% CI, 16.0-19.4).

Because more Dibao households were selected in the sample, we weighted the data by sample proportions. The results of weighted data produced only minor changes. In the baseline survey, 29.5% (95% CI, 28.3-30.8) of the studied population had depressive symptoms, while 17.8% (95% CI, 16.5-19.2) had severe depressive symptoms. The percentages of participants with depressive symptoms in the two follow-ups were lower than in the baseline survey. In the first follow-up, 27.0% (95% CI, 25.4-28.6) of the participants had depressive symptoms, and 17.2% (95% CI, 15.5-19.1) had severe depressive symptoms. In the second follow-up, 26.6% (95% CI, 24.3-29.0) of the participants had depressive symptoms, and 14.8% (95% CI, 13.0-16.7) had severe depressive symptoms.

### The relationship between health insurance and depression

**Figure 1:**
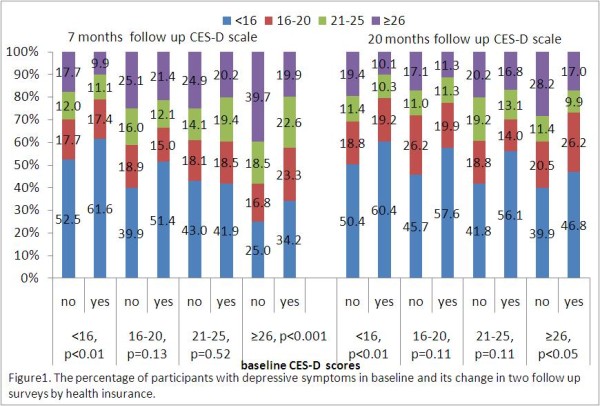
The percentage of participants with depressive symptoms in baseline and its change in two follow up sutveys by health insurance.

In the first follow-up survey, the percentage of participants with depressive symptoms significantly increased in the group without health insurance. When analyzing participants who did not have depressive symptoms in the baseline survey (CES-D scale scores <16), those without health insurance had a significantly higher rate of having depressive symptoms in follow-up surveys. In contrast, among those who had severe depressive symptoms in the baseline survey (CES-D scale scores ≥ 26), 39.7% of those who had no health insurance still had severe depressive symptoms in the first follow-up, while the rate was much lower (19.9%) for those who had health insurance. Similar trends appeared in the follow-up interview 20 months after the baseline survey (Figure 1).

In the analyses adjusted for baseline depressive level, when compared with the participants who had health insurance in the baseline and the first follow-up surveys. As shown in Table 3, participants without health insurance were more likely to have severe depressive symptoms (OR, 1.61; 95% CI, 1.31-1.98). Similarly, after adjusting for baseline depressive symptoms, city, sex, age, education, marital status, employment, economic status (Dibao), major life events, physical exercise, drinking behavior, BMI, and chronic disease in the previous four weeks, participants without health insurance were more likely to have severe depressive symptoms in both the baseline and first follow-up surveys compared with their counterparts (OR, 1.40; 95% CI, 1.08-1.80). This relationship was also found when comparing the baseline and second follow-up surveys. After adjusting for baseline depression and all the socioeconomic and personal factors, participants without health insurance were more likely to have severe depressive symptoms both in the baseline and second follow-up surveys, compared with their counterparts (OR, 1.89; 95% CI, 1.37-2.61). Moreover, participants who had insurance at baseline but lost the insurance by the second follow-up (OR, 2.09; 95% CI, 1.44-3.04) were more likely to have severe depressive symptoms.

**Table 3:** **Association between health insurance status and CES-D scale scores**^**a**^

	**7-month follow up CES-d, OR (95%CI)**	**P value**	**20-month follow up CES-d, OR (95%CI)**	**P value**
Ordinal regression adjusted with baseline CES-D				
Non-insurance	1.61 (1.31-1.98)	<.001	2.11 (1.59-2.80)	<.001
Insurance to None	1.22 (0.85-1.75)	.29	2.15 (1.51-3.06)	<.001
Non to Insurance	1.02 (0.70-1.49)	.91	1.57 (1.22-2.03)	<.001
insurance	1		1	
Ordinal regression adjusted with baseline CES-D, city, sex, ethnicity , age, education, marriage, employment, Dibao, life events.				
Non-insurance	1.44 (1.12-1.86)	.005	1.92 (1.39-2.65)	<.001
Insurance to None	1.21 (0.84-1.73)	.31	2.09 (1.45-3.00)	<.001
Non to Insurance	0.94 (0.65-1.37)	.76	1.45 (1.09-1.94)	.01
insurance	1	.	1	.
Ordinal regression adjusted with all above plus physical exercise, drink, smoke, BMI and 4-week disease				
Non-insurance	1.4 (1.09-1.82)	.01	1.89 (1.36-2.62)	<.001
Insurance to None	1.22 (0.85-1.76)	.28	2.1 (1.44-3.05)	<.001
Non to Insurance	0.92 (0.63-1.35)	.67	1.45 (1.09-1.95)	.01
insurance	1	.	1	.

A The OR reflects the cumulative probabilities of CES-D scale scores ≥26 vs CES-D scale scores 25 or less.

Table 4 presents the impact each factor had on depression in the follow-up surveys. Health insurance status, baseline depression level, Dibao, education, and BMI had significant impacts on whether a participant had depressive symptoms in the first follow-up. In contrast, only health insurance, baseline depression status, and Dibao had significant impacts on depression in the second follow-up. Compared with non-Dibao family participants, people from Dibao families were more likely to have severe depressive symptoms.

**Table 4:** The multivariate ordinal regression analysis for depression by health insurance status and background variables among adults in Gansu, China

		**7-month follow up CES-d, OR (95% CI)**	**P value**	**20-month follow up CES-d, OR (95% CI)**	**P value**
insurance	Non-insurance	1.40 (1.09-1.82)	.01	1.89 (1.36-2.62)	<.001
	Insurance to None	1.22 (0.85-1.76)	.28	2.10 (1.44-3.05)	<.001
	Non to Insurance	0.92 (0.63-1.35)	.67	1.45 (1.09-1.95)	.01
	insurance	1.00		1.00	
CES-D Baseline	<16	1.00		1.00	
	16-20	1.63 (1.24-2.15)	.001	0.92 (0.69-1.23)	.60
	21-25	1.67 (1.26-2.22)	<.001	1.16 (0.85-1.58)	.35
	≥26	2.38 (1.84-3.09)	<.001	1.54 (1.15-2.06)	.004
city	Lanzhou	0.98 (0.79-1.21)	.83	1.10 (0.86-1.40)	.44
	Baiyin	1.00	.	1.00	.
sex	Male	1.22 (0.95-1.55)	.12	1.13 (0.86-1.49)	.38
	Female	1.00	.	1.00	.
Ethnicity	Han	1.23 (0.81-1.87)	.32	0.88 (0.56-1.40)	.59
	Minority	1.00	.	1.00	.
age	16-35	0.99 (0.58-1.68)	.97	0.69 (0.39-1.23)	.21
	36-45	1.29 (0.80-2.08)	.29	0.87 (0.52-1.44)	.58
	46-55	1.25 (0.84-1.86)	.27	0.81 (0.53-1.26)	.35
	56-65	1.11 (0.77-1.60)	.58	1.05 (0.72-1.54)	.79
	≥66	1.00	.	1.00	.
education	primary school or less	2.08 (1.36-3.19)	.001	1.14 (0.74-1.76)	.56
	Junior high school	1.58 (1.11-2.27)	.01	1.04 (0.72-1.50)	.84
	High school	1.61 (1.13-2.29)	.01	1.03 (0.71-1.49)	.86
	College or more	1.00	.	1.00	.
marriage	Married	0.76 (0.46-1.24)	.27	0.59 (0.30-1.18)	.13
	Divorced	1.32 (0.76-2.29)	.32	1.04 (0.50-2.17)	.92
	Never-married	1.00	.	1.00	.
employment	Retired	1.04 (0.74-1.49)	.81	0.87 (0.60-1.26)	.45
	Unemployed	0.99 (0.76-1.30)	.95	1.00 (0.74-1.35)	.99
	employed	1.00	.	1.00	.
Dibao	Yes	1.54 (1.25-1.89)	.00	1.31 (1.05-1.64)	.02
	No	1.00	.	1.00	.
Life events	No	1.03 (0.72-1.48)	.85	1.04 (0.73-1.48)	.84
	1	1.18 (0.77-1.81)	.45	1.04 (0.68-1.61)	.85
	≥2	1.00	.	1.00	.
Physical activity	Yes	0.89 (0.72-1.10)	.29	0.99 (0.79-1.24)	.93
	no	1.00	.	1.00	.
drink	No	1.08 (0.73-1.60)	.71	1.08 (0.67-1.76)	.74
	Yes	1.00	.	1.00	.
smoke	No	1.00 (0.78-1.29)	.99	0.92 (0.70-1.22)	.56
	Yes	1.00	.	1.00	.
BMI	<19	1.67 (1.15-2.42)	.01	1.03 (0.69-1.54)	.88
	19-24	1.15 (0.90-1.48)	.27	0.85 (0.65-1.12)	.25
	≥25	1.00	.	1.00	.
4-weeks disease	No	0.81 (0.63-1.04)	.10	1.16 (0.87-1.53)	.31
	acute disease in 4 weeks	1.02 (0.61-1.69)	.94	1.20 (0.77-1.87)	.42
	acute disease > 4 weeks	0.68 (0.25-1.82)	.44	1.37 (0.67-2.81)	.39
	Chronic	1.00	.	1.00	.

## Discussion

This study assesses the impact of insurance on the development of depression. The results indicate that the prevalence of depression is high in northwest China, and both health insurance and poverty (measured by Dibao) have long-term influences on depression. These findings suggest that China’s Basic Health Insurance system is a significant protective factor against depression. This research provides further empirical evidence to justify the current Chinese primary health reform strategy that seeks to increase access to Basic Health Insurance in the general population. Nonetheless, there has been a worldwide systematic discrimination against mental disorders, which resulted in an exclusion of mental disorders from some social and private health insurance, regardless of economic achievement and stage of development. Examples of this tendency include the U.S. [33], some European countries [34], and China [35]. The rapid socioeconomic transition and the consequential traditional culture change in China had significantly increased the depression among the Chinese elderly and adult population [4,36]. Growing evidence suggests that Chinese people tend to view mental illness as a reflection of one's "inability to deal with social stress", "interpersonal conflict", or "personality deficits" [37,38]. Depression literacy may conceal the real situation [39]. In comparison, resources for mental health are scarce and unequally allocated [40]. The WHO reported that the treatment gap for severe mental disorders was 35–50% for developed countries and 76–85% for low- and middle-income countries [41]. Chinese policymakers should value the importance of mental health and invest greater resources in the future. Accordingly, China needs to reconsider its health care and service delivery system to accommodate the rapidly increasing issue of mental health. Specifically, China may need to increase mental health insurance coverage and encourage more non-psychiatric hospitals to offer psychiatric services.

The possible reason that may explain the impact of health insurance on reducing the risk of having depression is that obtaining insurance coverage can increase one’s sense of security while reducing the financial and psychological stresses created by medical treatment. Further studies are needed to indentify more reasons that may explain the relationship between health insurance and depression in China.

Survey data indicate that those who had no insurance at baseline but acquired coverage at the second follow-up still faced a higher risk of depression. This finding possibly results from the fact that data collection was prematurely conducted after the initiation of the new health insurance policy. The Basic Health Insurance scheme was launched in the summer of 2007 but was officially put into practice in the surveyed area in September. The second follow-up survey was performed between December 2007 and January 2008, and 636 participants were new insurance enrollees, comprising up to 33.7% of the sample, which was significantly higher than the 7.4% (n = 164) participants reported at the first follow-up. The percentage of participants without insurance dropped from 52.7% at the first follow-up (December 2006) to 23.6% at the second follow-up. Thus, the majority of newly enrolled participants acquired insurance quite recently during the survey. However, it is unlikely that they had any chance of using their insurance at the time of the survey. Thus, the impact of insurance may not be apparent in the data.

This study shows that there is a high prevalence of depression in Northwest China. When 21 score was used as the cutoff point, and the positive predictive value (PPV) was 55%, the prevalence of depression in northwestern Chinese cities was 14.6-16.2%. This rate was higher than that for mood disorders in general (6.7%) [4] and depression specifically among rural residents over 55 years of age (6%) [5]. This high prevalence might partially be linked to the location in which the survey was conducted. A previous population-based epidemiological study reported that the depression prevalence is significantly higher in northwestern part of China compared with the eastern and northern parts of the country [36].

Poverty is another important risk factor for depression. In addition to the demographic and socioeconomic factors, we also considered the effects of several other indicators, including major family or personal events, economic poverty (Dibao), personal health behaviors, and physical health status (diseases within the previous four weeks), all of which are factors closely related to depression according to previous studies. The three most significant predictors of depression are health insurance status, baseline depressive status, and poverty (Dibao). All other factors showed no long-term predictive relationship with depressive symptoms. In addition, prior studies proved that poverty and its associated psychosocial stressors, such as violence, unemployment, and insecurity, were correlated with the onset of adult mental disorders [42]. Another study showed that 70% of patients with mental illness within the lowest income group did not receive treatment due to their disadvantaged financial situation [43]. The government should give priority to poverty-stricken counties and provide them with essential financial assistance.

Due to the complexity of the topic, many other factors might interfere with the research conclusions. For instance, employment status is likely to be an important intervening factor in the relationship between health insurance and depression, as 94.9% of the people with insurance were enrolled in the employment-based BHIS. A previous study demonstrated a significant association between employment and self-reported state of health [44]. The presumed impact of health insurance enrollment on depression may be weakened by the factor of employment. However, no significant correlation between employment status and depression is found in this study, which indicates that the correlation between health insurance and depression is independent of employment status.

The quality of the data collected from the survey was satisfactory. The household, instead of the individual, comprised the survey sampling unit. Households were required to have at least one member 16 years of age or older to participate. Because the majority of the surveyed households had two members older than 16 years of age, the likelihood of each adult responding to the depression questions in each household was presumably 50%. According to this sampling design, the chance of each participant of the baseline depression survey being followed up in the successive surveys was also 50%. The actual follow-up rates of the study were 54.4% and 46.3% at the two follow-ups, which were reasonably acceptable given this design. Furthermore, because sample loss was random, the list-wise approach was employed to deal with missing data [45].

### Limitations and future trends

This study had several limitations. First, we did not include variables reflecting whether all participants had access to mental health services and to what extent they utilize the available services. Health insurance in China is still at a nascent level of development and does not provide full coverage for mental health treatment and rehabilitation. Second, we did not consider the effects of variables regarding the somatic expression of mental disorders and information concerning patients’ conditions provided by doctors. As discussed above, the Chinese tend to deny depression or express it somatically; neurosis is one such example [7]. Most likely, people may be more willing to talk to their physicians about such issues, especially when somatic problems are developed as a result of psychological stresses, because primary health care is largely covered by existing health insurance programs. Although the results of this study indicate a significant relationship between health insurance and depression, this hypothesis can only be confirmed to a certain extent. Further research is clearly needed. Third, the CES-D scale is a screening tool, not a clinical diagnostic tool, and the reference PPV is adopted from the elderly population. Consequently, the depression prevalence estimate may be higher than it actually is. Fourth, consideration of socioeconomic status was not thorough in this study. For instance, using Dibao to benchmark income and using unemployment and retirement to measure employment status may be taken into account in future research to more rigorously investigate the impacts of income and occupation. Finally, despite the advantage of keeping the sample intact, the list-wise approach employed in the study results in a loss of information. Thus, it may be favorable for future studies to employ other methods to deal with missing data.

## Conclusion

In communities of northwestern Chinese cities, people without Basic Health Insurance have a higher risk of depression than those with health insurance.

## Endnotes

^a^The number also included those who were enrolled in the Government Insurance Scheme (GIS) and Labor Health Insurance, which have been replaced by new insurance offerings in the studied areas since 2005. Some people continue to use this term for convenience, which included 64 people.

^b^More people were newly enrolled in the second follow-up survey than in the first follow-up survey because Lanzhou initiated the pilot operation of the URBMI in September 2007^34^.

## Competing interests

The authors declare no competing interests.

## Authors’ contributions

ZQ was primarily responsible for the study design, initiating and writing this report of the survey data as well as ensuring accurate data analysis. XZ applied for funding and participated in the survey design. XW was involved in the data collection. XZ, DT, XW, JG, FX and CL-WC were involved in the discussion for the writing of the manuscript and provided comments on the paper. All authors read and approved the final manuscript.

## Funding

This research is sponsored by the Project 985 fund of Beijing Normal University.

## Other support

In the process of data collection, the Department of Civil Affairs in Gansu province offered great support in assistance with sampling and household interviews. Dr. Ruiping Xin from Northwest Normal University organized the selection of interviewers and the data collection.

## Pre-publication history

The pre-publication history for this paper can be accessed here:

http://www.biomedcentral.com/1471-244X/12/151/prepub
